# MOM/GA-Based Virtual Array for Radar Systems

**DOI:** 10.3390/s20030713

**Published:** 2020-01-28

**Authors:** Kamel Sultan, Haythem Abdullah, Esmat Abdallah, Hadia El-Hennawy

**Affiliations:** 1 Electronics Research Institute, El-Nozha Elgededa, Cairo 12622, Egypt; haythm_eri@yahoo.com (H.A.); esmataa2@hotmail.com (E.A.); 2 Electronic and Communication Department, Faculty of Engineering, Ain shams University, Cairo 12622, Egypt; helhennawy@ieee.org

**Keywords:** virtual antenna array, automotive radar, long range radar, medium range radar, planar antenna array, flat shoulder shape, power divider

## Abstract

This paper introduces a novel antenna array synthesis for radar systems based on the concept of a virtual antenna array (VAA) and the method of moments/genetic algorithm (MoM/GA) synthesis method. The VAA concept is applied to both scanning and fixed radiation pattern arrays. The proposed VAA is introduced to simultaneously support the medium-range radar (MRR) and the long-range radar (LRR) with beam width ±7° for LRR and ±37° for MRR. The proposed VAA is distinguished by its minimum number of antenna elements, simple feeding network, high efficiency, and gain, but all of these are at the expense of a large aperture antenna size compared to the planar antenna array (PAA). The VAA has the ability to have the feeding network and the radiating elements on the same layer, as compared to the multilayer PAA. The newly proposed concept is analyzed and verified analytically and experimentally. Two orthogonal (16 elements) VAAs are designed to operate in the frequency range from 23.55 to 24.7 GHz and to support a flat-shoulder shape (FSS) radiation pattern for LRR/MRR. The antenna was fabricated and tested experimentally, and good agreements between the simulated and measured results were noticed. The proposed VAA is introduced to solve the problems of large size, low isolations, low efficiency, feeding network, low resolution, and small coverage range for the antenna arrays of automotive radars. The proposed antenna array is introduced for automotive radar applications at 24 GHz.

## 1. Introduction

Automotive systems are considered to be one of the world’s largest economic industries. With the advent of automotive radars, the automotive industry has adapted its industry to the new appearing technology [1,2]. Automotive radars make driving more comfortable, stable, and safe. The role of radar is to measure the range, speed, and direction of surrounding objects. Furthermore, with suitable identification algorithms, radar can discriminate between objects, and it controls vehicle action according to the type of object facing the radar system. The automotive radars are classified according to their operating range: long-range radar (LLR) (10–250 or 300 m), medium-range radar (MRR) (1–100 m), and short-range radar (SRR) (0.15–30 m); here, both LLR and MRR are required to detect forward obstacles [3,4,5,6].

Low profile, low cost, compact size, and ease of integration are the main concerns when solving the problems of automotive radar antenna design. In this part, we focus on the state of the art of radar antennas, especially 24 and 77 GHz automotive radar antennas [2,4,7,8,9,10].

In early radars for automotive applications that were first introduced in the 1970 s, huge and bulky antennas were utilized to meet necessary gains; these included as horn and parabolic antennas.

Nevertheless, these radars were a remarkable step in introducing the current professional radars ref. [11,12].

Thereafter, lens antennas became one of the famous antennas; they are the first choice for commercial automotive radar applications that provide a high directive beam and have a small size compared to horn and the aperture antennas. Lens antennas with a beam switching capability are better than a traditional phased antenna array due to the latter’s excessive metallic and phase shifter losses, specifically at higher frequency bands (24 and 77 GHz) [13]. In 2005, Colome et al. [14] introduced a cylinder lens fed by microstrip patches with a dual feed to increase the isolation between the transmitter and the receiver for bistatic radar at 24 GHz. This antenna achieves a gain of 15.3 dB and an HPBW of 21.30 and 37.80 for the E-plane and the H-plane, respectively. Based on the work in ref. [14], Weing et al. [15] introduced the same idea by placing a uniform series antenna array along the focus line of a lens. In the elevation (YZ) plane, the lens was fed by a column of a series of microstrip patch antennas. However, in the azimuth (XZ) plane, the receiving antenna consisted of a lens in conjunction with a uniform antenna array that was placed on the focal line of this lens.

On the other hand, to get away from the large size of the lens antennas, several types of planar antennas have been used. The merits of the planar antennas stem from their simple structure, low profile, ease of integration, and low manufacturing cost. The most common types of planar antennas that are used in the automotive radars are microstrip patch antenna arrays [16], grid antennas [9], slotted substrate integrated waveguide (SIW) [2], comb line [17] and cavity-backed [18]. In [19,20], an antenna array consisting of patches that were combined in series and/or in parallel arrangements to provide high directivity was introduced. Several papers have been introduced to address the problems of mutual coupling between elements and the radar cross section of the antenna array, as well as to provide high isolation between elements [21,22,23,24]. Vasanelli et al. [23] introduced a low radar cross-section antenna array based on the use of an artificial magnetic conductor (AMC) that was set around the series antenna to reduce the reflection in the direction of car fascia. The AMC had the capability to eliminate reflected waves from the antenna. Furthermore, in the last few months, Alibakhshikenari et al. used metamaterial unit cells [21] and metasurfaces [22,24] to reduce the mutual coupling between antenna elements to be compatible for radar applications.

Most of the radars in this side use two transmitters with two unique radiation patterns to give the long-range radar (LRR) and medium-range radar (MRR) modes. However, the performance of this system is not efficient for automotive radar due to the switching performance (time response and losses) between the two modes. Recently, Xu et al. [2,3] developed the idea of the shaped beam antenna that has been used in other applications such as satellites and radars to introduce a single antenna array that meets the requirements of LRR and MRR in automotive radar applications. The authors introduce two papers in this direction by using an SIW power divider to feed the planar patches array in [3] and to feed the SIW array slot in [2].

In this paper, the concept of a virtual antenna array (VAA) is utilized to reduce the number of elements in an antenna array and to provide high gain for LRR in comparison with the conventional planner antenna array (PAA). The basic concept of a VAA was introduced in [25,26,27,28]. The low complexity of the feeding network leads to a reduction of losses, which increases efficiency, thus achieving maximum gain. An analysis of the VAA is introduced, and a relationship between the proposed VAA and the PAA is set and verified by using the method of moments/genetic algorithm (MoM/GA) method [29]. The VAA is introduced to be simultaneously utilized for both LRR and MRR by offering a flat-shoulder shape (FSS) radiation pattern. The FSS radiation pattern is achieved with the use of an eight-by-eight VAA with the aid of the MoM/GA synthesis method. The complete antenna with a feeding network is simulated, fabricated and experimentally tested. The main contribution in this research is the flexibility and the reconfigurability of the synthesized radiation pattern in a compact form (VAA), as well as its simple feeding network.

## 2. Virtual Antenna Array Concept

**Figure 1:**
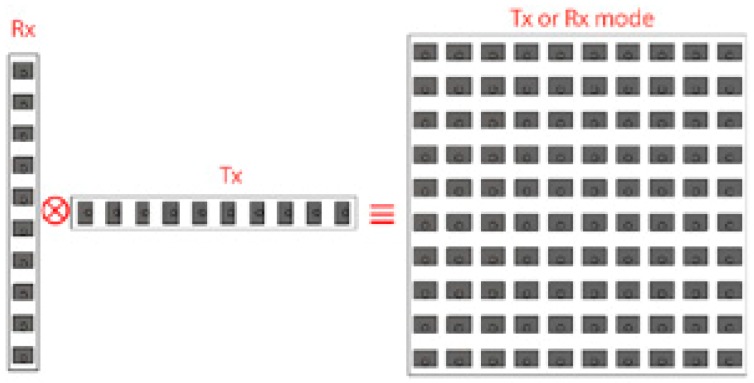
Virtual antenna array concept.

Suppose that an M element transmitting linear array distributed along the *x*-direction and an N element receiving linear antenna array distributed along the y direction. This distribution constitutes the basic building elements of the virtual antenna array. A VAA can be expressed by the convolution of the transmitter and the receiver antennas locations. Thus, a VAA can be generated by using sparse constituent arrays. This VAA can be much larger than an array of a conventional equivalent system; thus, the inherent performance of a VAA system will have more resolution than that of a traditional PAA. The concept of the VAA is illustrated in Figure 1. It clearly appears in radar applications where the received signal depends mainly on the multiplication of the radiation patterns for the transmitting and receiving antennas (two-way radiation pattern).

As is illustrated, the radiation pattern of the planar antenna shown on the right-hand side is equivalent to the multiplication of the radiation patterns of the two linear antenna arrays on the left-hand side of Figure 1.

The array factor of the transmitter linear antenna array is calculated according to Equation (1):

(1) $$AF_{T}\left(\theta ,\emptyset \right)=\overset{M}{\underset{m=1}{\sum }}a_{m}e^{jkd_{m}\sin\theta \cos\emptyset }$$

where $a_{m}$ is the *m*th complex excitation coefficient and θ and ∅ are the spherical polar and azimuth angles, respectively. The gain of the transmitting antenna is proportional to the square of the array factor such that:

(2) $$G_{T}\left(\theta ,\emptyset \right)=\alpha_{T}{\left|{\hat{AF}}_{T}\left(\theta ,\emptyset \right)\right|}^{2}$$

where ${\hat{AF}}_{T}\left(\theta ,\emptyset \right)$ is the normalized transmitter array factor and, similarly, the receiver array factor could be written as:

(3) $$AF_{R}\left(\theta ,\emptyset \right)=\overset{N}{\underset{n=1}{\sum }}a_{n}e^{jkd_{n}\sin\;\theta \;\sin\emptyset }$$

The receiver gain is also written as:

(4) $$G_{R}\left(\theta ,\emptyset \right)=\alpha_{R}{\left|{\hat{AF}}_{R}\left(\theta ,\emptyset \right)\right|}^{2}$$

where ${\hat{AF}}_{R}\left(\theta ,\emptyset \right)$ is the normalized receiving array factor.

Suppose that an object of radar cross-section σ is positioned in front of the transceiver system; then, the receiving power is calculated according to Equation (5) so that:

(5) $$P_{rV}=\frac{P_{tV}G_{R}\left(\theta ,\emptyset \right)G_{T}\left(\theta ,\emptyset \right)\sigma \lambda^{2}}{{\left(4\pi \right)}^{3}R^{4}}h\left(\theta ,\emptyset \right)$$

where $P_{rV}:$ received power in watts of the virtual antenna array; $P_{tV}:$ peak transmitted power in watts of the virtual antenna array; $G_{T}:$ transmitter gain; $G_{R}:$ receiver gain; λ: wavelength (m); σ: RCS of the target (m^2^); R: range between radar and target (m); and h(θ,∅): the channel response/coefficient for the wave impinging from the transmitter and reflected from the scatterer under investigation and then back to the receiving point.

In the case of normal radar (monostatic radar) operation where one antenna is utilized for both transmission and reception, the used antenna may be a planar array of the M × N elements of array factor equals:

(6) $$AF_{P}\left(\theta ,\emptyset \right)=\overset{M}{\underset{m=1}{\sum }}a_{m}e^{jkd_{m}\sin\theta \cos\emptyset }⊗\overset{N}{\underset{n=1}{\sum }}a_{n}e^{jkd_{n}\sin\;\theta \;\sin\emptyset }$$

where ⊗ denotes to the Kronecker product [30]. From Equations (1), (3) and (6), it can be noted that the array factor of the planar array in either receiving or transmitting modes equals the multiplication of the array factor of the transmitter and the array factor of the receiver of the virtual array. As such, the planar array factor is written as:

(7) $$AF_{P}\left(\theta ,\emptyset \right)=AF_{T}\left(\theta ,\emptyset \right)⊗AF_{R}\left(\theta ,\emptyset \right)$$

The gain of the array factor equals:

(8) $$G_{P}\left(\theta ,\emptyset \right)=\alpha_{P}{\left|{\hat{AF}}_{P}\left(\theta ,\emptyset \right)\right|}^{2}$$

where ${\hat{AF}}_{P}$ is the normalized array factor of the planar array.

It can be seen in Equations (2), (4), (6) and (8) that the gain of the planar antenna array could be written in terms of the gain of the virtual array transceiver as follows:

(9) $$G_{P}\left(\theta ,\emptyset \right)=G_{T}\left(\theta ,\emptyset \right)⊗G_{R}\left(\theta ,\emptyset \right)$$

In case of the planar array, the received power is calculated as follows:

(10) $$P_{rP}=\frac{P_{tP}G_{P}^{2}\left(\theta ,\emptyset \right)\sigma \lambda^{2}}{{\left(4\pi \right)}^{3}R^{4}}h\left(\theta ,\emptyset \right)$$

where $P_{rP}:$ received power in watts of the planar antenna array; and $P_{tP}:$ peak transmitted power in watts of the planar antenna array.

After comparing Equation (5) with Equation (10) and taking them into consideration with Equation (9), the following relation is held:

(11) $$P_{rP}=G_{P}\left(\theta ,\emptyset \right)⊗P_{rv}$$

This means that the received power that uses the planar antenna array is greater than the array factor of the virtual array by a factor of $G_{P}\left(\theta ,\emptyset \right).$

On other words, the VAA can be described in term of beam steering vector. The VAA is considered as the multiplication between the beam steering vectors of *Rx* and *Tx* (Figure 1) and can be expressed in the following matrix form:

(12) $$v\left(\theta ,\emptyset \right)={\left[\begin{array}{c} e^{jkd_{r1}\sin\theta \sin\emptyset }\;\\ e^{jkd_{r2}\sin\theta \sin\emptyset }\\ \vdots \\ \vdots \\ e^{jkd_{rN}\;\sin\theta \sin\emptyset }\; \end{array}\right]}_{Rx}⊗{\left[e^{jkd_{t1}\sin\theta \cos\emptyset },\;e^{jkd_{t2}\sin\theta \cos\emptyset },\;\ldots \ldots \ldots .,\;e^{jkd_{tM}\sin\theta \cos\emptyset }\;\right]}_{Tx}\;={\left[\begin{array}{cccc} e^{jk\left(d_{r1}\sin\emptyset +d_{t1}\cos\emptyset \right)\sin\theta } & e^{jk\left(d_{r1}\sin\emptyset +d_{t2}\cos\emptyset \right)\sin\theta } & \cdots & e^{jk\left(d_{r1}\sin\emptyset +d_{tM}\cos\emptyset \right)\sin\theta }\\ e^{jk\left(d_{r2}\sin\emptyset +d_{t1}\cos\emptyset \right)\sin\theta } & e^{jk\left(d_{r2}\sin\emptyset +d_{t2}\cos\emptyset \right)\sin\theta } & \ldots & e^{jk\left(d_{r2}\sin\emptyset +d_{tM}\cos\emptyset \right)\sin\theta }\\ \vdots & \vdots & \ldots & \vdots \\ e^{jk\left(d_{\mathrm{rN}}\sin\emptyset +d_{t1}\cos\emptyset \right)\sin\theta } & e^{jk\left(d_{\mathrm{rN}}\sin\emptyset +d_{t2}\cos\emptyset \right)\sin\theta } & \ldots & e^{jk\left(d_{\mathrm{rN}}\sin\emptyset +d_{tM}\cos\emptyset \right)\sin\theta } \end{array}\right]}_{N\times M}$$

where v is the steering vector of VAA, *d_r_* is the distance between elements in *Rx*, and *d_t_* is the distance between elements in *Tx*.

From Equation (12), it can be noted that the steering vector of VAA is equivalent to the conventional beam steering of PAA.

Then what is the benefit of the virtual array? The reply to this question is introduced in the following section.

## 3. Antenna Array Synthesis and the Verification of the VAA

**Figure 2:**
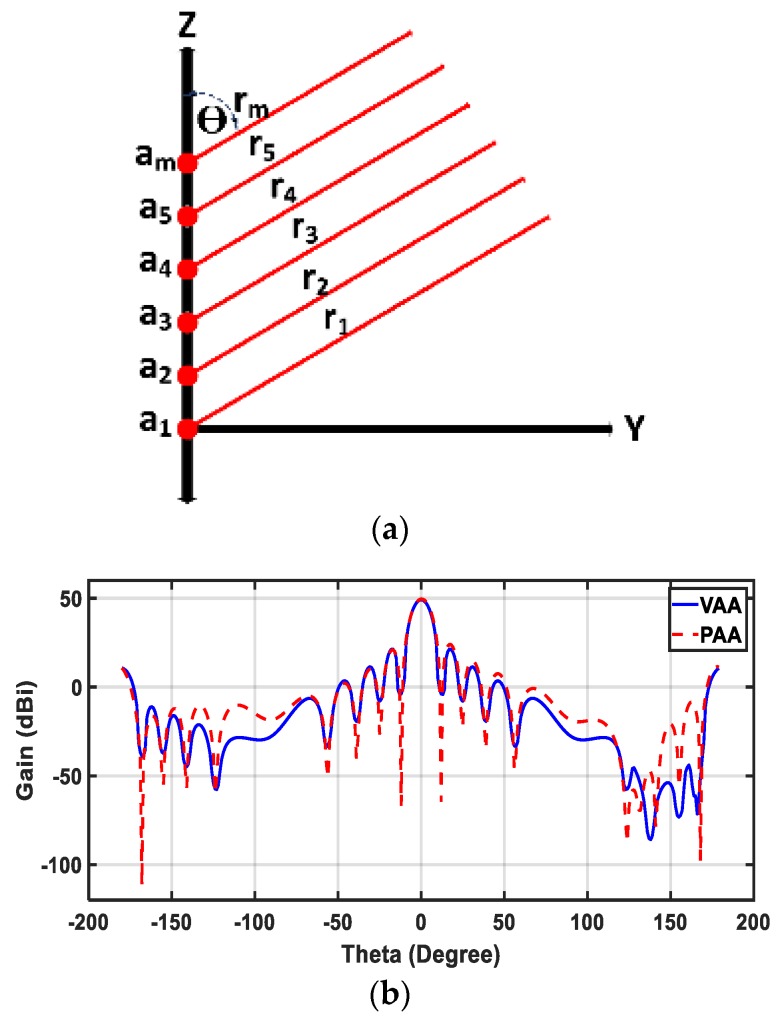
(**a**) Linear isotropic antenna array and (**b**) comparison between radiation pattern of the planar antenna array (PAA) and the virtual antenna array (VAA).

The radiation pattern of linear antenna arrays is synthesized by using several techniques: analytical techniques, optimization techniques, and semi-analytical techniques. In this work, the MoM/GA method [29], a semi-analytical technique, was utilized to synthesize the two columns of the virtual array to mimic the planar array. Consider the configuration of an isotropic sources antenna array as shown in Figure 2a. The isotropic sources are arranged along the *z*-axis with equal distance and uniform shape (*r_i_* is the distance between elements and far field observation point, *a_n_* is the excitation coefficient of the nth element, and θ is the angle between the antenna array axis and the far field axis). The number of the antenna array elements is defined according to the integration between the method of moments (MoM) and the genetic algorithm (GA) to give a reduction in the number and the spacing of antenna array elements. The MoM is used to give the excitation coefficients, while the GA is utilized to give the best locations of the antenna array elements with the minimum tolerance. The MoM/GA method summarizes the synthesis problem with the following equation:

(13) $${\left[Z\right]}_{M\times M}{\left[I\right]}_{M\times 1}={\left[V\right]}_{M\times 1}$$

where the elements of the matrix ${\left[Z\right]}_{M\times M}$ are given by:

(14) $$z_{mn}=\overset{\pi }{\underset{0}{\int }}e^{j\left(d_{n}-d_{m}\right)kcos\left(\theta \right)}d\theta$$

and the elements of the vector ${\left[V\right]}_{M\times 1}$ are given by:

(15) $$V_{m}=\overset{\pi }{\underset{0}{\int }}AF_{d}\left(\theta \right)e^{-jkd_{m}\cos\left(\theta \right)}d\theta$$

The excitation coefficients $a_{n}$ are determined by solving the linear system of Equation (13), where $a_{n}$ are the elements of the matrix ${\left[I\right]}_{M\times 1}$ and ${\left[I\right]}_{M\times 1}={\left[a_{1},\;a_{2},\;a_{3},\;\ldots \ldots ,\;a_{M}\right]}^{T}.$

In order to get the same received power for both the planar and the virtual array, the number of elements for the transmitter and the receiver array should be adjusted so that:

(16) $$G_{P}\left(\theta ,\emptyset \right)=\sqrt{G_{Tnew}\left(\theta ,\emptyset \right)⊗G_{Rnew}\left(\theta ,\emptyset \right)}$$

where $G_{Tnew}\left(\theta ,\emptyset \right)$ and $G_{Rnew}\left(\theta ,\emptyset \right)$ are the gain of the transmitter and the receiver of the virtual MIMO, respectively. It is worth mentioning that the length of the virtual array is kept greater than the length of the planar array in order to keep the same gain. However, the benefit of the virtual array is to the ability to minimize the number of elements, thus reducing the feeding network size and, consequently, reducing the losses and increasing the gain. Another benefit is that the total area of the VAA is small compared to the PAA.

In this section, the application of the MoM/GA method realizes the equivalence of both the VAA and the PAA.

By using Equation (16) and by applying the MoM/GA method to the VAA with only 18 elements in the transmitting array and 18 elements in the receiving array, one can have the same equivalent radiation pattern of the PAA with 100 elements of the transmitting planar array and another 100 elements of the receiving array. The PAA radiation pattern is the desired radiation pattern to be achieved when using the virtual antenna array $AF_{d}\left(\theta \right)$. $AF_{d}\left(\theta \right)$ is discretized within the domain from θ=0–π into *n* values. The values of *n* are estimated in such a way that all variations of the radiation pattern are covered by discretization points. In our case, 20 points between the two null points are enough. Thus, the integration in Equation (15) is numerically implemented M times. Each time, the integration is evaluated with a different $d_{m}$ value according to the location of each of the M antenna elements in the array in order to fill the ${\left[V\right]}_{M\times 1}$ matrix of Equation (13). Similarly, the matrix ${\left[Z\right]}_{M\times M}$ is filled by using Equation (14) after discretization with the same number of points. Finally, Equation (13) is solved with the Gaussian elimination method [31] in order to get the excitation coefficient matrix ${\left[I\right]}_{M\times 1}$. The resulting excitation coefficients are symmetric around the center line of the array, so the excitation coefficients of the first nine elements of the proposed VAA, in this case, were [0.15, 0.257, 0.363, 0.462, 0.571, 0.667, 0.781, 0.868, and 1.0]. The spacing between elements were chosen according to the genetic algorithm to be 0.5 wavelengths. The radiation pattern of the virtual array was calculated by using the resulting excitation coefficients. Figure 2b shows a comparison between the VAA and the PAA radiation patterns in the two orthogonal planes because the two antennas are symmetric.

In this experiment, the virtual antenna array of 36 total number of elements was equivalent to the 200 elements planar antenna array. Sometimes the antenna array of radar systems adds another device, a circulator, to separate between the transmitting and the receiving signals to avoid the use of two planar antennas. However, even with this case, the number of elements of the VAA was still competitive (36 virtual array/100 planar array) in addition to the size, complexity, and losses in the separator device. The low number of array elements in the virtual antenna array reduced the complexity and the losses of the feeding network to a great extent. This allowed for a high efficiency and gain of the VAA compared to the PAA. However, these benefits came at the expense of the total aperture size of the VAA compared to the PAA with the circulator. Furthermore, the PAA still needed another layer for the feeding network. Additionally, the VAA still competed for the use of two planar arrays for the transmission and reception.

In case of the scanning antenna array, the required control circuits for the VAA was very low compared to the PAA array.

In this section, antennas without feeding are introduced to illustrate the concepts of passive feeding and active feeding, as well as the relationships between the VAA and the PAA. The next section introduces an application of the VAA in automotive systems. In most automotive sensors (automotive radars), designers introduce several sensors that surround the vehicle with broadside radiation. Each sensor services a fixed direction, e.g., LRR and MRR in front of the vehicle and SRR in the sides of the vehicle. Some others use scanning sensors to identify the direction of the target in front of the radar. In the next section, a fixed beam sensor is introduced, but the scanning sensor is made straight forward by removing the feeding network and instead applying control circuits.

## 4. Antenna Design for MRR and LRR

After verifying the VAA concept and comparing its performance relative to the PAA, it was the time to use this concept for synthesizing an FSS radiation pattern. In addition to the aforementioned merits of the VAA, the use of the VAA to implement an FSS pattern adds another feature—the simultaneous operation of MRR and LRR.

This section is divided into three subsections to provide the full design for an antenna array that takes the merits of the VAA and the FSS pattern. First, the FSS pattern is demonstrated and the MoM/GA method is applied to get the excitation coefficients of the antenna array that should be applied to the elements of the array to get the FSS pattern. Second, a proposed feeding network based on the resulting excitation coefficients is simulated, fabricated, and verified. The last sub-section is devoted to the feeding of the VAA array by using the designed feeding network.

### 4.1. Proposed FSS Radiation Pattern

In order to mitigate the problem of switching between the radiation pattern of the MRR system and the LRR system, the two systems are merged into one system. This acquires the synthesis of a radiation pattern that simultaneously suits the two applications. To synthesize this appropriate radiation pattern, assume that LRR and MRR have the ranges of $R_{L}\;\mathrm{and}\;R_{m},$ respectively, such that $R_{L}=kR_{m},$ where *k* is a constant number that relates the long-range with the medium range.

Assume that the minimum received power at the radar is constant in the two modes $P_{rm}$. Let $G_{T}^{L}\;\mathrm{and}\;G_{T}^{M}$ represent the transmitting antenna gains for LRR and MRR, respectively. Additionally, assume, typically, that $R_{m}=100\;m$ and *k* = 3. The minimum received power is expressed as [2]:

(17) $$P_{rm}=\frac{P_{t}G_{t}G_{r}\sigma \lambda^{2}}{{\left(4\pi \right)}^{3}R^{4}}=\frac{P_{t}G_{t}^{M}G_{r}\sigma \lambda^{2}}{{\left(4\pi \right)}^{3}R_{m}^{4}}=\frac{P_{t}G_{t}^{L}G_{r}\sigma \lambda^{2}}{{\left(4\pi \right)}^{3}{\left(kR_{m}\right)}^{4}}$$

By the simple manipulation of Equation (17), the gain difference $G_{d}$ between the two operating modes can be calculated as:

(18) $$\left[G_{d}\right]=\left[G_{t}^{L}\right]-\left[G_{t}^{m}\right]=40\;\log_{10}\;k$$

**Figure 3:**
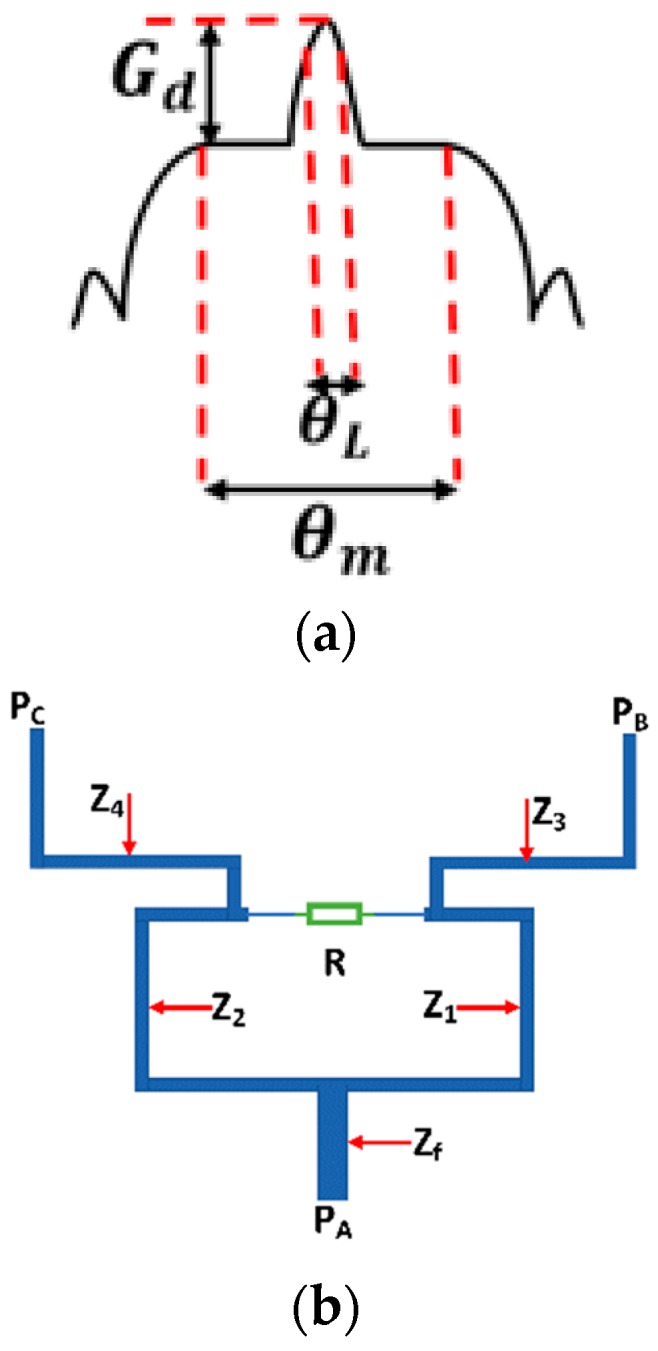

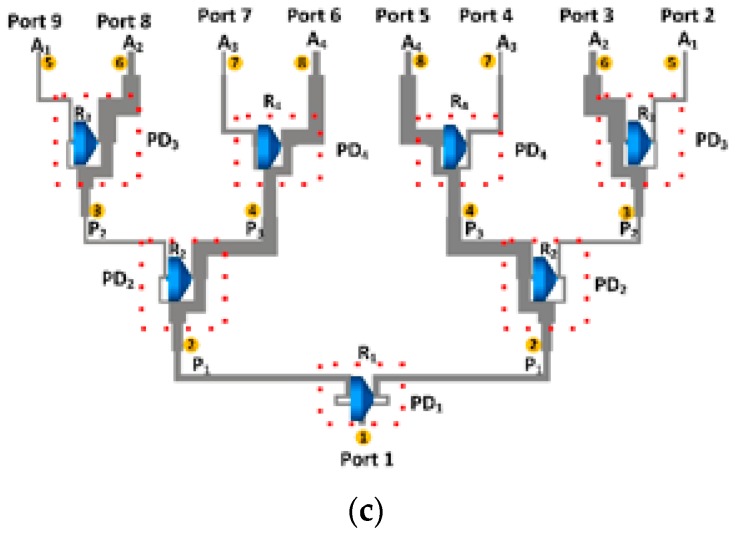
(**a**) Proposed flat-shoulder shape (FSS), (**b**) reference power divider, and (**c**) proposed feeding network.

The expected ideal radiation pattern of the proposed system is shown in Figure 3a. This shape is called the flat-shoulder shape (FSS), and, in our experiment, the half power angle for LRR was $\theta_{L}\;=\;15^{{}^{\circ}}$ at −3 dB relative to the maximum of the radiation pattern, and the beam width for MRR was $\theta_{m}=\pm \;40^{{}^{\circ}}$ at −3 dB relative to the $G_{d}$ level.

The radiation pattern (FSS) in Figure 3a was applied to the MoM/GA method as a desired radiation pattern. The synthesis process resulted in excitation coefficients that guaranteed the occurrence of the FSS pattern when exciting an eight element antenna array whose elements were half-wavelength spaced from each other. The details of achieving these excitation coefficients via a feeding network based on Wilkinson power dividers is demonstrated in the following subsection.

### 4.2. Feeding Network of the VAA

**Figure 4:**
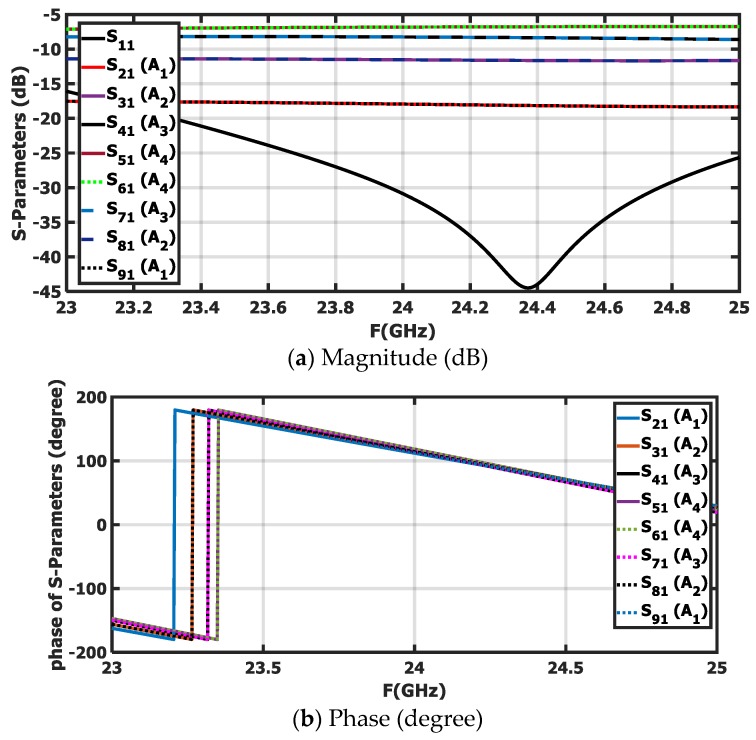
S-parameters of power dividers.

Because the FSS pattern is considered a broadside radiation pattern, the phase shift between elements should vanish. After applying the MoM/GA method, it was noticed that the excitation coefficient pattern was symmetric around the center of the array. The left-handed elements were excited by −17.122 and −11.04 dB, and the right-handed elements were excited by −7.642 and −6.38 dB. The configuration of the feeding network is shown in Figure 3c. The feeding network consisted of one main equal Wilkinson power divider (PD_1_) and two sets of one-to-four power dividers. The set of one-to-four power dividers consisted of three unequal power dividers (PD_2_, PD_3_, and PD_4_). It was proposed to implement the power dividers PD2, PD3 and PD4 as having the ratio of P1, P2 and P3, respectively. P1, P2, and P3 have ideal or proposed magnitudes of −3, −10.838, and −3.788 dB and simulated magnitudes of −3.21, −10.62, and −4.67 dB, where the $P_{1}=\frac{P_{{\mathrm{out}|}_{\;\mathrm{at}\;\mathrm{circle}\;2}}}{P_{{\mathrm{in}|}_{\;\mathrm{at}\;\mathrm{circle}\;1}}},{\;P}_{2}=\frac{P_{{\mathrm{out}|}_{\;\mathrm{at}\;\mathrm{circle}\;3}}}{P_{{\mathrm{in}|}_{\;\mathrm{at}\;\mathrm{circle}\;1}}}$, and $P_{3}=\frac{P_{{\mathrm{out}|}_{\;\mathrm{at}\;\mathrm{circle}\;4}}}{P_{{\mathrm{in}|}_{\;\mathrm{at}\;\mathrm{circle}\;1}}}$ are the ratios between output powers at its circle and the input power. The feeding network was implemented on a Rogers RO4003 substrate with a dielectric constant of 3.38 and a thickness of 0.2 mm. Quarter wavelength transformers and stepped impedance matching were utilized to achieve the required excitation coefficient at the 24 GHz frequency. The power dividers in the feeding network were based on the Wilkinson power divider shown in Figure 3b. All the required equations to design Wilkinson power dividers were introduced in [32]. The impedances of power divider lines were calculated from Equation (19) and are listed in Table 1 after optimization. The Wilkinson power divider was selected due to its wide bandwidth and high isolation between its ports. The matching between the power divider stages and the antenna elements was achieved by using quarter wavelength transformers. All the simulated S-parameter magnitudes and the phase of the proposed power dividers are depicted in Figure 4. Table 2 illustrates the comparison between the ideal synthesized excitation coefficients and the simulated output of the feeding network. It can be noticed that the simulated results are very close to the ideal results. Equation (19) is as follows:

(19) $$Z_{1}=Z_{f}\sqrt{\frac{1+F^{2}}{F^{3}}},\;Z_{2}=F^{3}Z_{1},\;Z_{3}=Z_{f}/F,\;Z_{4}=Z_{f}F,\;R=2Z_{f},\;F=\frac{P_{B}}{P_{C}}$$

where $Z_{f}$ is the characteristic impedance of feed line, *R* is the impedance between branches of power divider, and F is the factor of the power ratio between ports.

### 4.3. Implementation of the VAA Antenna

**Figure 5:**
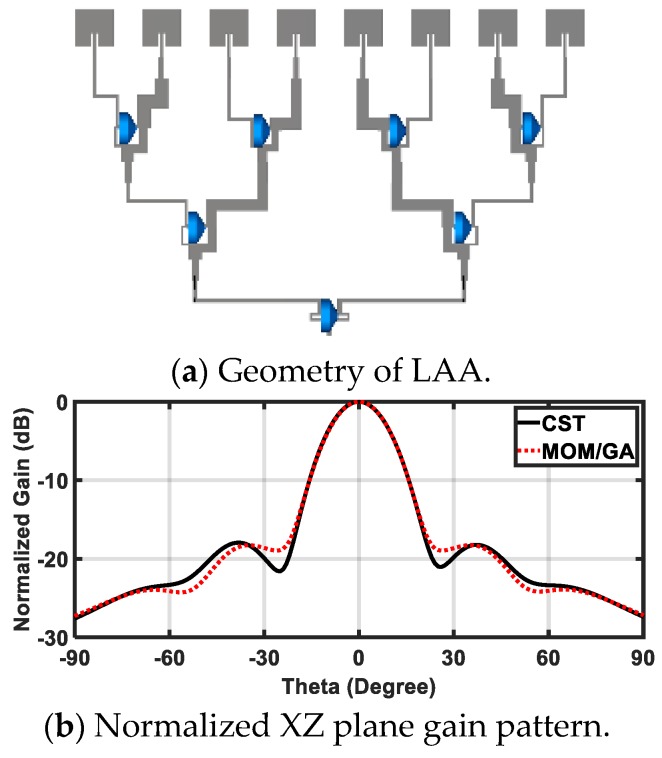
Linear antenna array.

**Figure 6:**
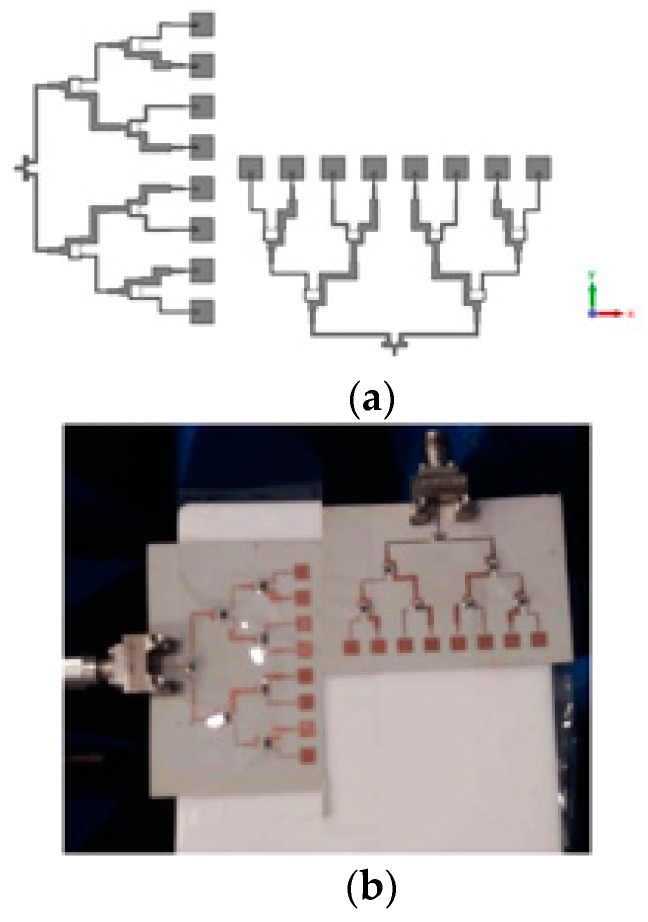
(**a**) VAA configuration and (**b**) photo of VAA.

**Figure 7:**
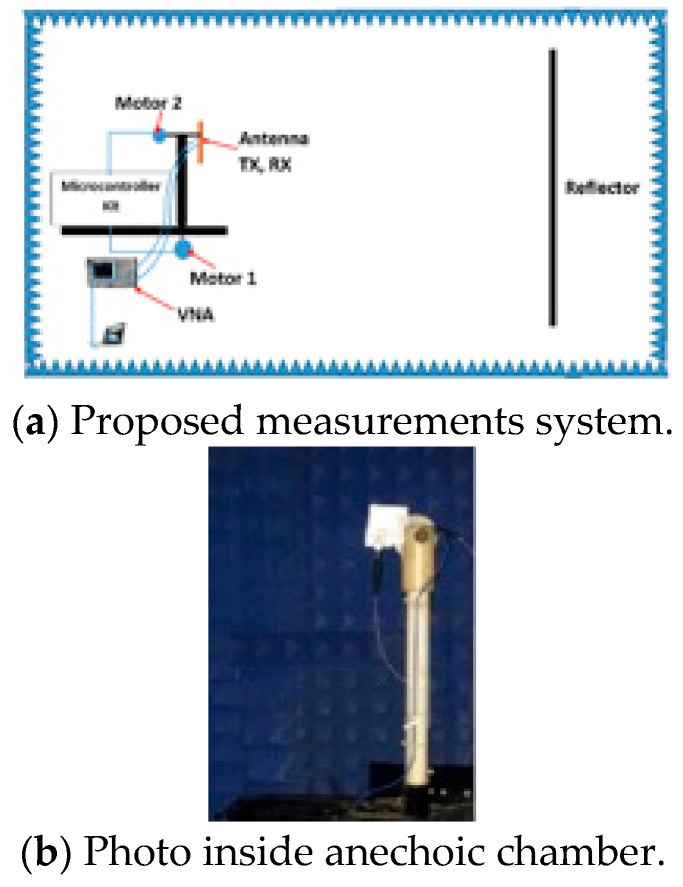
Setup system for VAA measurements.

**Figure 8:**
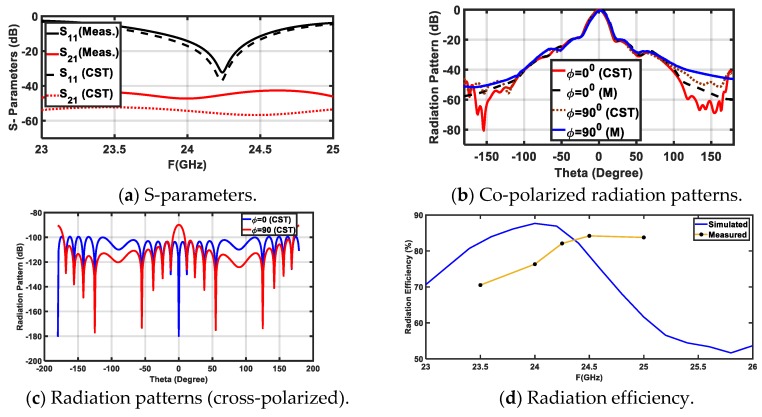
Simulated and measured results of VAA.

In this section, the proposed MRR and LRR antenna design is introduced. Figure 5a shows the geometry of the rectangular patch linear antenna array with a half a wave length as the distance between the elements (patch length = 3.2 mm, patch width 3.45 mm, and the inset feed = 1.1 mm from the patch edge). The matching between the antenna patches and the power divider was introduced based on the quarter wave length transformer between the output impedance of PD3 and PD4 (inserted as *Z*_3_ and *Z*_4_ for each power divider in Table 1) and the input impedance of the antenna. The antenna array was fed by the proposed power divider to give the proposed FSS radiation pattern. The FSS pattern was achieved as shown in Figure 5b, where a good agreement between the simulated FSS pattern from CST and the required FSS pattern from the MoM/GA method can be noticed. We noticed that the FSS pattern had two-levels to serve MRR and LRR at the same time. The concept of the VAA was applied to the proposed antenna, as shown in Figure 6. Figure 6 shows the geometry and prototype of the VAA concept by fixed transmitter and receiver antenna that were perpendicular to each other. The VAA was fabricated on the same substrate of the feeding network shown in Figure 6b. The radiation pattern measurement setup is depicted in Figure 7. The VAA was fixed on a rotating setup that had an azimuth and an elevation rotation facility. The two ports of the VNA were used for transmission and reception. A 1 m^2^ plate was placed in front of the VAA antenna. A wave at 24 GHz emanated from the horizontal antenna and impinged on the plate. The back scattered wave was received by the vertical array. By recording the received signal at different angles, one could predict the VAA radiation pattern. It was noticed that the radiation pattern of our VAA in the two orthogonal planes achieved an HPBW = $\pm 7^{{}^{\circ}}$ (at −3 dB) for LRR and a beam width of −19 dB = $\pm 40^{{}^{\circ}}$ for MRR, as shown in Figure 8b,c. Figure 8a shows the simulated and measured S-parameters, as well as the radiation pattern, of the VAA. Good agreements between the simulated and measured results were observed. The radiation efficiency of the antenna array is shown in Figure 8d, where it can be observed that the average radiation efficiency from simulated and measured results was about 86% through the interested band.

Table 3 shows the comparison between our proposed VAA for automotive radars and the antennas that were introduced in the literature. As far as we know, only two papers in the literature have combined the LRR and MRR modes [2,3], but these papers had a complicated feeding SIW network with antenna sizes of 13.2$\lambda_{0}$ × 5.1$\lambda_{0}$ and 3.9$\;\lambda_{0}$ × 7.9$\lambda_{0}$, excluding the feeding network size for the antenna in [3] and antenna in [2], respectively. Our design had a small size compared to the aforementioned antennas and achieved the same radiation properties. Furthermore, the proposed design of the VAA concept was able to reduce the number of antenna elements to eight patches for *Tx* and eight patches for *Rx* compared with the 60 patches used in [3] and the 96 slots used in [2]. Furthermore, the proposed design achieved the requirements of LRR and MRR beam angles in the E-plane and the H-plane. Otherwise, the automotive radar antennas that have been introduced in the literature have had a large size, a low gain and a huge number of elements. The benefits of the proposed VAA can be summarized as its simple structure, low profile, printing on a single layer, low number of elements, high gain, high efficacy and simultaneous serving of MRR and LRR. In Table 3, the VAA is compared to literature papers where the proposed antenna achieved the best performance in beam width, size and performance in addition to simultaneously serving MRR and LRR.

.

## 5. Conclusions

A novel concept in the design of an automotive radar antenna system is introduced in this paper. The VAA concept is designed to have a simple, highly isolated and a highly efficient antenna array that competes with the PAA in most of its characteristics. The proposed concept has been analyzed and verified by using an analytical solution in conjunction with the MoM/GA method to get the optimum characteristics of the VAA compared to the PAA antenna. The work ended up with a design of a VAA antenna that has a feeding network that is based on a cascaded network of Wilkinson power dividers on the same substrate; the overall dimensions of this VAA are 30 × 48 × 0.2 mm^3^. The antenna has an FSS radiation pattern that simultaneously supports both MRR and LRR applications. The proposed antenna achieves the required gain and HPBW that simultaneously give a high resolution to MRR and LRR. The experimental results agree well with simulated results in terms of radiation patterns and reflection coefficients. In this work, it has been proven that the VAA can achieve the same gain and beam width of the PAA with low losses and a low number of elements.

## Figures and Tables

**Table 1:** Power divider branches impedances (optimization values).

PD	*Z_f_*	*Z*_1_	*Z*_2_	*Z*_3_	*Z*_4_	*R*
1	50	70.7	70.7	50	50	100
2	26.2	76.1	33.46	68.6	28.5	135
3	26.79	76.217	33.311	66.494	25.77	68
4	33.31	65.1	39.94	55.94	27.5	135

**Table 2:** Excitation coefficients.

	Synthesis (Ideal)		Simulated (CST)	
	Amplitude (dB)	Phase	Amplitude (dB)	Phase
A1	−17.122	00	−17.72	00
A2	−11.04	00	−11.6	1.80
A3	−7.642	00	−8.19	1.20
A4	−6.38	00	−6.88	3.70

**Table 3:** Comparison between proposed antenna and antennas in literature.

Ref.	F (GHz)	Radar	Antenna Description	HPBW		Gain (dBi)	BW (GHz)	Size	$\varepsilon_{r}$
				E Plane	H Plane				
**[2]**	77	LRR/MRR	SIW (6 × 16)	±7.5°, ±40°	-	21.7	2.9	(WOFN)	2.2
								3.9λ_0_ × 7.9λ_0_	
**[3]**	77	LRR/MRR	Microstrip + SIW (6 × 10)	±7.5°, ±40°	-	20	9.5	(WOFN)	2.2
								13.2λ_0_ × 5.1λ_0_	
**[4]**	80	LRR	VP	±7°	-	25.6	1.5	(WFN)	3.38
			(16 × 16) RX, (2 × 16) TX					5.9λ_0_ × 6.4λ_0_	
**[8]**	77	LRR	RP (8 × 8)	±9°	-	20	1	5.9λ_0_ × 6.4λ_0_	3.38
**[9]**	24	SRR	Grid 33 elements	7°	90°	13.8	6	1.44$\lambda_{0}$ × 11.7$\lambda_{0}$	3
**[10]**	23.7	SRR	Patches with mushroom	10°	15°	11	1	2.9$\lambda_{0}$ × 5.3$\lambda_{0}$	3.38
**[23]**	77	LRR	10-element Rectangular	20°	-	4	-	(WOFN)	3
			series, AMC					9.7λ_0_ × 3.6λ_0_	
**[33]**	77	LRR	RP (5 × 14)	±10°	-	20.5	1.5	8.1$\lambda_{0}$ × 2.95$\lambda_{0}$	11.8
**[34]**	77	LRR	VRP (8 × 18)	4.8°	18.3°	20.8	1	17.96$\lambda_{0}$ × 7.7$\lambda_{0}$	2.2
**[35]**	77	LRR	VRP (2 × 1)	±15°	-	18.5	2.4	10.3$\lambda_{0}\times$ 10.3$\lambda_{0}$	3.38
**[36]**	77	LRR	RP (32 × 32) (MC)	10°	-	24.7	0.7	10.8$\lambda_{0}$ × 19.2$\lambda_{0}$	3.38
**This Work**	24.1	LRR/MRR	16 RP VAA	±7°, ±38°	±7°, ±38°	22	1.15	(WFN)	3.38
								3.6λ_0_ × 2λ_0_	

RP: rectangular patch; VRP: varying rectangular patch; MC: microstrip comb-line; WOFN: without feeding network, which means that the total size of feeding network was not available in this paper; WF: includes the size of the feeding network; VAA: virtual antenna array; LRR: long range radar; MRR: medium range radar; AMC: artificial magnetic conductor.
